# Fast-Track in Minimally Invasive Gynecology: A Randomized Trial Comparing Costs and Clinical Outcomes

**DOI:** 10.3389/fsurg.2021.773653

**Published:** 2021-11-11

**Authors:** Shahzia Lambat Emery, Philippe Brossard, Patrick Petignat, Michel Boulvain, Nicola Pluchino, Patrick Dällenbach, Jean-Marie Wenger, Georges L. Savoldelli, Benno Rehberg-Klug, Jean Dubuisson

**Affiliations:** ^1^Department of Pediatrics, Gynecology, and Obstetrics, Geneva University Hospitals and University of Geneva, Geneva, Switzerland; ^2^University of Geneva, Geneva, Switzerland; ^3^Department of Anesthesiology, Division of Anesthesiology Clinical Pharmacology, Intensive Care and Emergency Medicine, Geneva University Hospitals and Faculty of Medicine, University of Geneva, Geneva, Switzerland

**Keywords:** fast-track, laparoscopic hysterectomy (LH), hospital costs, hospital stay, pain assessment, post-operative morbidity

## Abstract

**Study Objective:** Evaluate the effects of a fast-track (FT) protocol on costs and post-operative recovery.

**Methods:** One hundred and seventy women undergoing total laparoscopic hysterectomy for a benign indication were randomized in a FT protocol or a usual care protocol. A FT protocol included the combination of minimally invasive surgery, analgesia optimization, early oral refeeding and rapid mobilization of patients was compared to a usual care protocol. Primary outcome was costs. Secondary outcomes were length of stay, post-operative morbidity and patient satisfaction.

**Main Results:** The mean total cost in the FT group was 13,070 ± 4,321 Euros (EUR) per patient, and that in the usual care group was 3.5% higher at 13,527 ± 3,925 EUR (*p* = 0.49). The FT group had lower inpatient surgical costs but higher total ambulatory costs during the first post-operative month. The mean hospital stay in the FT group was 52.7 ± 26.8 h, and that in the usual care group was 20% higher at 65.8 ± 33.7 h (*p* = 0.006). Morbidity during the first post-operative month was not significantly different between the two groups. On their day of discharge, the proportion of patients satisfied with pain management was similar in both groups [83% in FT and 78% in the usual care group (*p* = 0.57)]. Satisfaction with medical follow-up 1 month after surgery was also similar [91% in FT and 88% in the usual care group (*p* = 0.69)].

**Conclusion:** Implementation of a FT protocol in laparoscopic hysterectomy for benign indications has minimal non-significant effects on costs but significantly reduces hospital stay without increasing post-operative morbidity nor decreasing patient satisfaction.

**Clinical Trial Registration:** www.ClinicalTrials.gov, identifier: NCT04839263.

## Precis

The implementation of a fast-track protocol in laparoscopic hysterectomy for benign indications has some benefits with no associated risks.

## Introduction

The concept of “enhanced recovery” was developed by cardiologists in the 1950s to improve patient rehabilitation after myocardial infarction. In 1995, this concept was extended to colon surgery by Kehlet et al. (1), who developed a perioperative multimodal strategy currently known as “fast-track” (FT) surgery or “enhanced recovery after surgery” (ERAS). This innovative concept includes the combination of minimally invasive surgery, analgesia optimization, early oral refeeding, and rapid mobilization of patients. The objective is to reduce pain and organic dysfunction induced by surgical stress (2). This strategy facilitates patient recovery and comfort while decreasing morbidity and hospital stay (3, 4).

FT surgery is a multimodal approach that requires a close and well-codified multidisciplinary collaboration among surgeons, anesthetists, and the nursing team. The patient is at the center of this concept and is considered to play a major role in the success of FT surgery. Appropriate patient information & education and high patient motivation are necessary (5–11).

A recent review of the medical literature showed only marginal use of FT protocols in gynecological surgery (12). Observational studies of patients undergoing laparoscopic hysterectomy have indicated that FT protocols appear to be effective in reducing hospital stay without increasing post-operative morbidity. However, no randomized trials have been conducted to evaluate the effectiveness of a FT protocol in gynecological laparoscopic surgery for benign indications (13–22).

The main objective of this trial was to compare the effectiveness of a FT protocol in laparoscopic hysterectomy for benign indications vs. usual care in terms of costs. Length of stay, post-operative morbidity, and patient satisfaction were also compared in between the two protocols.

## Methods

All patients undergoing total laparoscopic hysterectomy for a benign indication, with or without oophorectomy, in the Department of Pediatrics, Gynecology and Obstetrics of the Geneva University Hospitals from September 2015 to January 2020 were offered participation in this randomized controlled trial.

The exclusion criteria were (1) the requirement for an additional surgical procedure, such as prolapse repair or urinary incontinence, because a prolonged operative time could compromise early patient discharge and (2) the inability to speak French because the patients were required to complete their data collection logbook in French. Eligible patients were given information about the study, and patients who agreed to participate provided written informed consent. The study protocol was approved by the Cantonal Ethics Committee of Geneva.

Consenting patients were randomized into two groups: the FT group (intervention group) and the usual care group (control group). The randomization list was created by a computer program using randomly permuted blocks of different sizes (two and four). Sealed, opaque, numbered envelopes were prepared, and patients were included consecutively by our research nurse.

**Figure 1:**
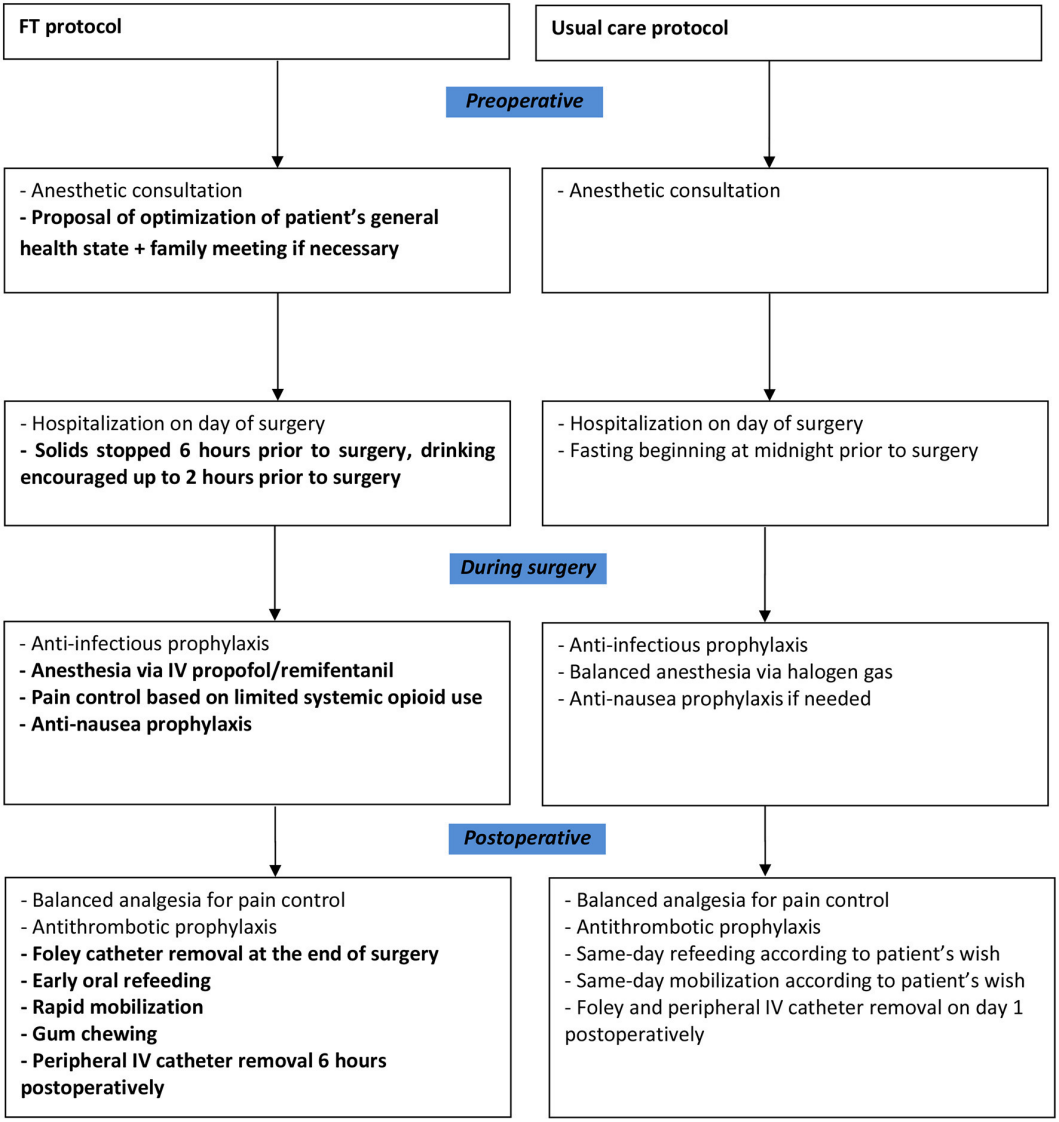
FT and usual care protocol descriptions. Items in the FT protocol that differ from those in the usual care protocol are highlighted in bold. FT, fast-track; IV, intravenous.

Our FT protocol was established according to the guidelines for pre-operative and intraoperative care in gynecologic surgery (23). In this protocol, there was a more complete pre-operative evaluation of the patients general health (Figure 1). Obese patients were advised to lose weight and smokers were advised to stop smoking. The mean Katz Index of Independence in Activities of Daily Living (Katz ADL) was evaluated [6 = high (patient independent) to 0 = low (patient very dependent)] and a meeting with the patient's family was proposed if needed. Patients in both groups were admitted on the day of surgery. In the usual care protocol, fasting was required as of midnight the day prior to surgery. In the FT protocol, solids were stopped 6 h prior to surgery and patients were encouraged to drink clear liquids up to 2 h prior to surgery. At anesthetic induction, both groups received antibiotic prophylaxis with 2 g of intravenous (IV) cefazolin.

In the FT protocol, general anesthesia was maintained using a total IV technique (propofol/remifentanil). In the usual care protocol, a balanced inhalational anesthesia technique (sevoflurane/sufentanil) was used. In the FT group, intraoperative pain control was based on limited systemic opioid use and patients prophylactically received 4 mg of IV dexamethasone and 4 mg of IV ondansetron to prevent post-operative nausea and vomiting (PONV). In the usual care protocol, only patients known to have motion sickness and those with a medical history of PONV received anti-nausea prophylaxis.

In the FT group, the urinary Foley catheter was removed at the end of the laparoscopic procedure. Four hours post-operatively, the patients underwent oral refeeding, were mobilized, and were given gum to chew. Six hours post-operatively, the peripheral IV catheter was removed. In the usual care group, the urinary Foley catheter and peripheral IV catheter were removed on day 1 post-operatively. The patients were allowed same-day refeeding and mobilization 6 h post-operatively according to their desire.

Patients in both groups received antithrombotic prophylaxis with low-molecular-weight heparin 6 h post-operatively. In both groups, post-operative pain was controlled using balanced analgesia with a combination of non-steroidal anti-inflammatory drugs and acetaminophen that was supplemented with oral morphine if needed.

In both groups, the surgical procedure consisted of a standardized total laparoscopic hysterectomy. The patients were placed in the lithotomy position with the arms alongside the body. A urinary Foley catheter was inserted into the bladder at the beginning of the procedure. Pneumoperitoneum was created using a Veress needle. The intra-abdominal pressure was lowered to 12 mmHg during the procedure. One umbilical or supra-umbilical port (depending on the uterus size) was inserted for the optic laparoscope (5- or 10-mm diameter depending on the surgeon's preference) and three accessory ports were used for the standard laparoscopic instruments (5-mm diameter). A uterine manipulator (HOHL; Karl Storz, Tuttlingen, Germany) was used to mobilize the uterus during the dissection. The surgical steps were standardized according to the European Society for Gynecological Endoscopy (24). The uterus was removed through the vagina. When the surgeon was unable to retrieve a large uterus transvaginally in one piece, morcellation techniques were used preferably using transvaginal cold morcellation to transabdominal power morcellation. The vaginal cuff was sutured with multifilament absorbable sutures using intracorporeal knots. Senior surgeons engaged in regular surgical activity performed all the surgical procedures.

All patients received a logbook to evaluate pain, refeeding, and mobilization during their hospital stay. Pain was evaluated based on a visual analog scale (VAS) ranging from 0 (no pain) to 10 (worst possible pain).

The same standard criteria for hospital discharge were applied in both groups: normal physical examination, no fever, effective non-opioid oral analgesia, normal feeding, absence of PONV, independent mobility, and patient consent. Patients went home with step 1 analgesics according to the World health Organization Analgesic Ladder (25).

The Katz ADL was used to evaluate problems in performing activities of daily living and home care was organized if needed. In both groups, planned outpatient post-operative follow-up examinations were performed on days 7 and 30.

The primary outcome was costs and secondary outcomes were length of stay, post-operative morbidity, and patient satisfaction.

The economic evaluation was conducted from a societal perspective and covered hospital inpatient surgical care costs and ambulatory costs. Ambulatory costs were further divided into hospital-related care costs (consultations at the Accident and Emergency (A&E) Department and hospital readmission) and community costs (community health & patients informal caregiver's loss of production costs). The economic evaluation covered the period from inpatient surgical care hospitalization to 28 days post-operatively. Resource inputs were divided into two main categories: (1) hospital inpatient surgical costs (inpatient surgical care hospitalization) and (2) ambulatory costs further divided into (2a) hospital-related costs (A&E Department consultations and hospital readmission) and (2b) community costs (community health & social costs and caregiver's loss of production costs). All resource inputs attributable to each patient's hospital costs were collected using a computerized hospital information system developed by the University Hospitals of Geneva (26). It must be pointed out that, in Switzerland, surgical costs are fixed in function of the surgical procedure and accounted for as such. Patients recorded community costs in a logbook containing the community health and social invoices and caregivers number of absent working days. Caregiver's loss of production was extrapolated via Switzerland's median wage per working day (27). All unit costs were expressed in Euros (EUR).

Hospital stay was retrieved from the computerized patient record. Morbidity during the first post-operative month was monitored via patient consultations at the A&E Department. Patient satisfaction was evaluated on their day of discharge and at their 1-month post-operative follow-up visit using a few questions, based on a three-point Likert scale, regarding their satisfaction with the care they received.

The sample size of 170 patients (FT protocol, *n* = 85; usual care protocol, *n* = 85) was calculated to be able to show a difference, with a type I error of 5% and a power of 90%, of one-half of the standard deviation (SD) in continuous measures (cost analysis). Data were analyzed using an intention-to-treat approach. Patients remained in the group to which they were initially allocated at the time of randomization.

Descriptive statistics were used to report patient characteristics. The hospital stay, costs, and VAS pain scores were compared between the groups using a *t*-test. Morbidity during the first post-operative month (number of emergency consultations) and patient satisfaction were compared between the groups using Fisher's exact test. Analyses were conducted using Stata and R software, and a *p*-value of <0.05 was considered statistically significant.

## Results

**Figure 2:**
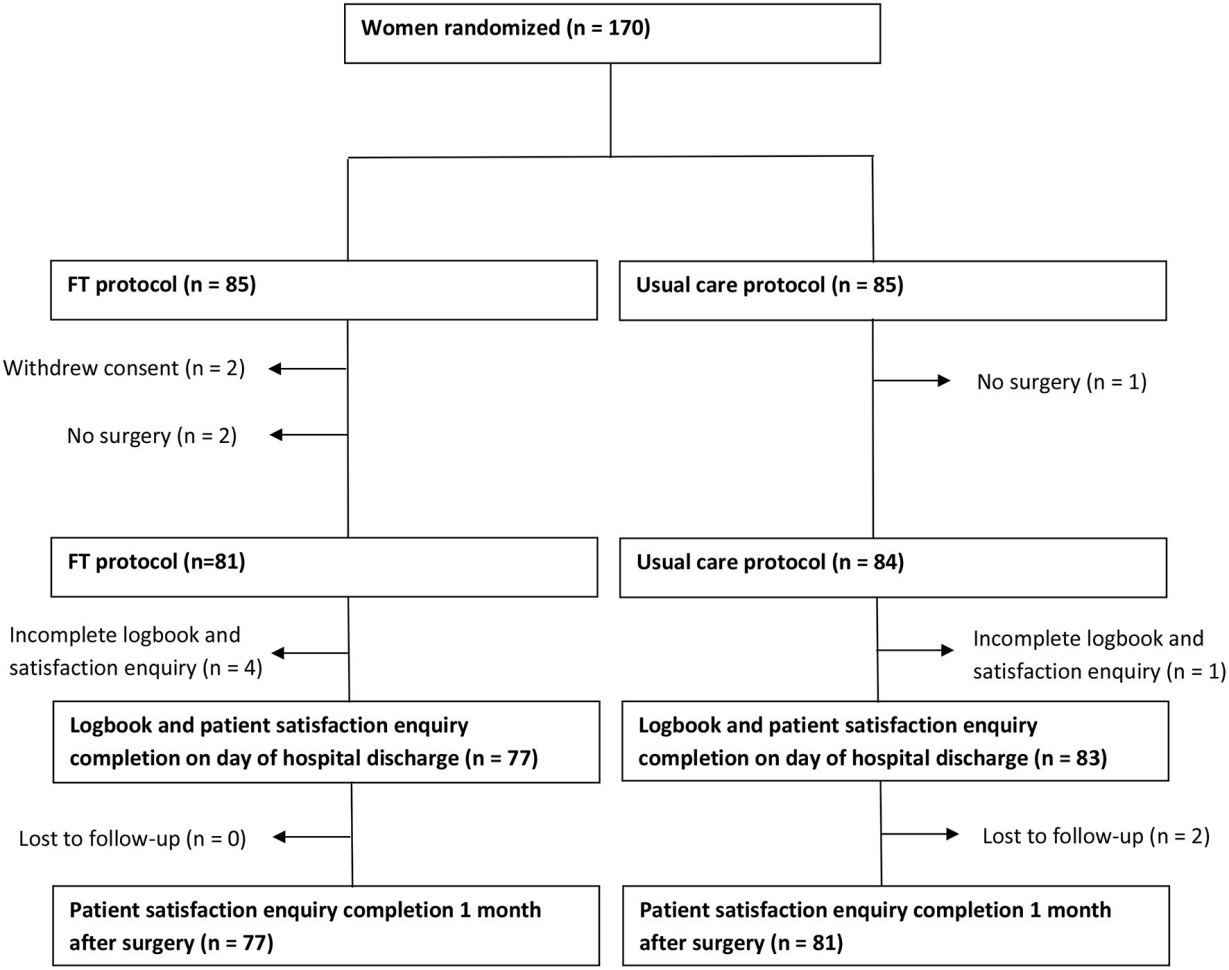
Study flowchart. FT, fast-track.

From September 2015 to January 2020, 170 patients (85 in each group) were enrolled in this study. Five patients did not complete the study: two decided not to undergo surgery, one underwent uterine artery embolization (no further surgery), and two withdrew their consent to participate (Figure 2).

Patients in both groups were similar in terms of age, body mass index, active smoking, comorbidities, and surgical indications (Table 1). The Katz ADL in both groups was 6, meaning that most of the patients in both groups were totally independent. The lowest score in both groups was 5. No smokers or obese patients in the FT group stopped smoking or lost weight before the intervention.

**Table 1:** Patients' baseline characteristics.

**Variable**	**FT group** **(*n* = 85)**	**Usual care group (*n* = 85)**
**Age, years**	46 ± 7	47 ± 6
**BMI, kg/m^2^**		
Normal (18.5–24.9)	41 (48.2%)	32 (37.6%)
Overweight (25.0–29.9)	22 (25.9%)	30 (35.3%)
Obese (≥30.0)	22 (25.9%)	23 (27.1%)
**Active smoking**	24 (28.3%)	22 (25.9%)
**Comorbidities**		
Diabetes	2 (2.4%)	4 (5.9%)
Hypertension	11 (12.9%)	12 (14.1%)
Hypercholesterolemia	8 (9.4%)	12 (14.1%)
Thromboembolic disease	5 (5.9%)	6 (7.1%)
Katz ADL^*^		
6	75 (88.2%)	73 (85.9%)
5	10 (11.8%)	12 (14.1%)
**Surgical indication**		
Myoma	60 (70.6%)	60 (70.6%)
Endometriosis/adenomyosis	22 (25.9%)	21 (24.7%)
Cervical dysplasia	2 (2.3%)	3 (3.5%)
Other	1 (1.2%)	1 (1.2%)

*Data are presented as mean ± standard deviation or n (%).*

*FT, fast-track; BMI, body mass index; Katz ADL, Katz Index of Independence in Activities of Daily Living.*

* *6 = high (patient independent) to 0 = low (patient very dependent)*.

The mean total cost in the FT group was 13,070 ± 4,321 EUR per woman, and that in the usual care group was 3.5% higher at 13,527 ± 3,925 EUR (*p* = 0.49). The hospital inpatient surgical costs were 12,507 ± 4,182 EUR in the FT group and 13,025 ± 3,829 EUR in the usual care group (*p* = 0.41). The total ambulatory costs during the first month after surgery were 621 ± 1,015 EUR in the FT group and 532 ± 1,081 EUR in the usual care group (*p* = 0.59). The hospital outpatient costs were 151 ± 329 EUR in the FT group and 98 ± 278 EUR in the usual care group (*p* = 0.28). The community costs were 469 ± 946 EUR in the FT group and 434 ± 1,053 EUR in the usual care group (*p* = 0.82).

The mean hospital stay in the FT group was 52.7 ± 26.8 h, and that in the usual care group was 20% higher at 65.8 ± 33.7 h (*p* = 0.006).

The mean VAS pain score on the day of surgery was 4.8 ± 3.0 in the FT group and 5.6 ± 2.8 in the usual care group (*p* = 0.08). On day 1 post-operatively, the mean VAS pain score was 3.8 ± 2.5 in the FT group and 4.1 ± 2.3 in the usual care group (*p* = 0.34).

We recorded one perioperative complication in the usual care group. A woman with two prior cesarean sections had a bladder perforation during dissection. It was diagnosed during surgery and surgically repaired during the same operation. The patient went home on post-operative day 3 with a Foley catheter that was left in the bladder for a total of 7 days. After removal of the Foley catheter, the patient did not suffer any urinary sequelae.

During the first post-operative month, we recorded 32 post-operative complications in the FT group and 36 in the usual care group (*p* = 0.78). The frequency and severity of complications were similar between the two groups according to the Clavien–Dindo classification (Table 2).

**Table 2:** Details of complications according to Clavien–Dindo classification.

	**FT group** **(*n* = 81)**	**Usual care group** **(*n* = 84)**	***p*** **-value**
**Any complication**	**32 (39.5%)**	**36 (42.3%)**	**0.78**
**Grade 1**	**11 (13.6%)**	**4 (4.8%)**	**0.09**
Important post-operative pain	1	0	
Scab fall	2	0	
Vaginal hematoma	3	1	
Fever of unknown origin	1	0	
Urinary retention	4	2	
Bladder perforation repaired during initial surgery	0	1	
**Grade 2**	**20 (24.7%)**	**32 (38.1%)**	**0.09**
Anemia	2	7	
Constipation	4	1	
Urinary tract infection	7	10	
Vaginal infection	5	7	
Vaginal hematoma infection	0	2	
Vaginal cuff abscess	1	1	
Vaginal cuff dehiscence	0	3	
Pulmonary embolism	1	0	
Left pelvic plexus thrombus	0	1	
**Grade 3**	**1 (1.2%)**	**0**	
Vaginal tear requiring surgery	1	0	
**Grade 4**	**0**	**0**	

*Data are presented as n (%) or n.*

*FT, fast-track*.

One patient in the FT group was diagnosed with pulmonary embolism 3 days after the operation despite post-operative antithrombotic prophylaxis with low-molecular-weight heparin. She was treated with rivaroxaban for a total of 3 months on an ambulatory basis. This patient was a 51-year-old smoker (20 pack-years) and had a 5-month history of palpitations that had not been investigated prior to surgery.

Only one complication required surgical revision. It was the case of a woman in the FT group. The uterus was vaginally morcellated with an iatrogenic 2 cm tear of the vagina. The patient presented with important post-operative bleeding on day 2 needing surgical suture. The rest of the post-operative period was uneventful and the follow-up examination on day 30 showed a total healing of the vagina with no sequelae.

Patient satisfaction on the day of discharge and at the 1-month post-operative follow-up visit was generally high, with no statistically significant differences between the groups in terms of pain management and medical or nursing follow-up (Table 3).

**Table 3:** Patient satisfaction on day of hospital discharge and at 1-month post-operative follow-up visit.

**Satisfaction**	**FT group**	**Usual care group**	***p*** **-value**
**On day of hospital discharge**	**(*n* = 77)**	**(*n* = 83)**	
Pain management	64 (83.1%)	65 (78.3%)	0.57
Medical follow-up	74 (96.1%)	74 (89.2%)	0.17
Nursing follow-up	72 (93.5%)	76 (91.6%)	0.87
**One month after surgery**	**(*n* = 77)**	**(*n* = 81)**	
Pain management	59 (76.6%)	62 (76.5%)	>0.99
Medical follow-up	70 (90.9%)	71 (87.7%)	0.69
Nursing follow-up	69 (89.6%)	75 (92.6%)	0.70

*Data are presented as n (%).*

*FT, fast-track*.

## Discussion

The results of our randomized trial confirm that, as in colorectal surgery, the implementation of a FT protocol in laparoscopic hysterectomy for benign indications is feasible.

In the cost analysis, our study showed no significant difference between the FT protocol and usual care in a laparoscopic setting. A study by Modesitt et al. (28) showed that 30-day total hospital costs were significantly decreased for both open procedures and minimally invasive procedures when implementing enhanced recovery in major gynecologic surgeries. The difference might be due to the fact that the duration of the hospital stay, which is the main driver of costs, is already short after laparoscopic hysterectomy for a benign indication in contrast to major gynecologic procedures.

In terms of length of stay, our results are in agreement with those found in other FT protocols in gynecological surgery. In a study by Kuster Uyeda et al. (29) on a FT protocol for perioperative care in general gynecological surgery, the hospitalization time was significantly reduced by 5.5 h. A possible explanation for the shorter hospital stay could have been a diminished post-operative pain; however, our study showed a non-significant reduction in the mean VAS pain score of 10% in the FT group compared with the usual care group on the day of surgery and no difference in the reported pain on post-operative day 1. Other studies have shown similar effects of FT protocols on pain, suggesting that the key to a reduced hospital stay is the well-codified multimodal approach in the FT protocol rather than one specific element (30).

A recent literature review by Scheib et al. (12) suggested that the use of a FT protocol in open or minimally invasive gynecologic surgery had a complication rate similar to that of usual care. In our study, post-operative morbidity was not significantly different between the two groups and most of the post-operative A&E Department consultations were for minor issues such as constipation, scab falls, and urinary tract infections.

One patient in the FT group was diagnosed with pulmonary embolism 3 days after her operation but she also had a history of palpitations that had not been investigated prior to surgery. This case confirms the importance of preventive health optimization prior to any surgery. One patient required surgical revision due to an iatrogenic 2 cm tear of the vagina after transvaginal cold morcellation. This case stresses the importance of morcellating under visual control using vaginal retractors to protect the genitourinary tract (31).

Most studies have shown that patient satisfaction is not altered in a FT setting. In a study on enhanced recovery implementation in cytoreduction, surgical staging, or pelvic organ prolapse surgery, Kalogera et al. (32) showed that 95% of patients rated their satisfaction with their perioperative care as excellent or very good. The results of our trial showed similar satisfaction with the provided care.

To our knowledge, this is the first randomized trial that compares a FT protocol to usual care in laparoscopic hysterectomy for benign indications. Most studies published to date either focused on FT protocols matched to historical controls and/or compared multiple surgical approaches (open, vaginal, and/or laparoscopic). The results of our study show a minimal benefit of routine implementation of a FT protocol in laparoscopic hysterectomy for benign indications. Our hypothesis is that minimally invasive surgery for hysterectomy already includes most components of a FT protocol, making the difference only marginal.

One limitation of our study is that our trial was not conducted in an ambulatory setting because in Europe, an overnight stay is more common after laparoscopic hysterectomy for benign indications than in the United States, where such patients commonly undergo same-day surgery (33). Nevertheless, by reducing length of stay, the implementation of a FT protocol could encourage to promote successful ambulatory surgery in Europe.

Another limitation of our study is that it only evaluated a laparoscopic approach for benign conditions. We believe that these results would be different if a FT protocol had been implemented in more complex situations, such as oncologic procedures necessitating open surgeries and/or complex treatments for endometriosis. Further randomized controlled trials evaluating FT protocols in those settings need to be conducted.

## Conclusion

Our results show that the implementation of a FT protocol in laparoscopic hysterectomy for benign indications has minimal non-significative effect on costs but significantly reduces hospital stay without increasing post-operative morbidity nor decreasing patient satisfaction. The results of our study show that routinely implementing a FT protocol in laparoscopic hysterectomy for benign indications has some benefits with no associated risks.

## Data Availability Statement

The raw data supporting the conclusions of this article will be made available by the authors, without undue reservation.

## Ethics Statement

All procedures involving human participants were in accordance with the ethical standards of the institutional and/or national research committee and with the 1964 Helsinki declaration and its later amendments or comparable ethical standards. This study was approved by the Cantonal Ethics Committee of Geneva https://www.ge.ch/document/liste-protocoles-soumis-2015 (CCER 15-103), approved on the 21st of July 2015. Informed consent was obtained from all individual participants included in the study.

## Author Contributions

All authors contributed to the study conception and design. Material preparation, data collection, and analysis were performed by SL and JD. The first draft of the manuscript was written by SL and all authors commented on previous versions of the manuscript. All authors read and approved the final manuscript.

## Funding

This study was supported by a grant from the Fondation Privée des Hôpitaux Universitaires de Genève.

## Conflict of Interest

The authors declare that the research was conducted in the absence of any commercial or financial relationships that could be construed as a potential conflict of interest.

## Publisher's Note

All claims expressed in this article are solely those of the authors and do not necessarily represent those of their affiliated organizations, or those of the publisher, the editors and the reviewers. Any product that may be evaluated in this article, or claim that may be made by its manufacturer, is not guaranteed or endorsed by the publisher.
